# Microbiological Comparison of Maxillary Sinus Rinses in Non-Odontogenic and Odontogenic Sinusitis of Primarily Endodontic Origin

**DOI:** 10.3390/jcm14144880

**Published:** 2025-07-09

**Authors:** Marta Aleksandra Kwiatkowska, Aneta Guzek, Dariusz Jurkiewicz, Iwona Patyk, Barbara Pajda, Piotr Rot

**Affiliations:** 1 Department of Otolaryngology and Oncological Laryngology with Division of Cranio-Maxillo-Facial Surgery, Military Institute of Medicine—National Research Institute, 04-142 Warsaw, Poland; 2 Department of Microbiology, Military Institute of Medicine—National Research Institute, 04-142 Warsaw, Poland; 3 Kuyavian-Pomeranian Pulmonology Center, 85-326 Bydgoszcz, Poland

**Keywords:** odontogenic sinusitis, microbiology, chronic rhinosinusitis, maxillary sinus, periapical lesion

## Abstract

**Objectives:** Odontogenic sinusitis (ODS) is common but frequently overlooked condition that differs from rhinogenic sinusitis (CRS) and should be suspected in each case of unilateral sinusitis. Clinical symptoms such as foul smell, congestion, rhinorrhea, and unilateral maxillary sinus opacification with overt dental pathology on radiological scans are more suggestive of ODS than CRS, but the distinctive microbiological flora are another clinical factor in diagnosis. The aim of this study was to compare the microbiological load of ODS and CRS and their clinical presentation for better disease recognition and its predisposing factors. **Methods:** Adult patients scheduled for endoscopic sinus surgery were included in the study. Clinical data and radiological images were analyzed. The otolaryngologist assessed nasal endoscopy for mucopurulence or edema in middle meatus or sinuses, whereas dental specialist confirmed or ruled out the dental cause. Microbiological samples were collected after endoscopic maxillary antrostomy. After irrigation with 0,9% saline, the aspirated rinse was collected into sterile sets and sent for culturing. **Results:** The study group consisted of 84 patients, 55 with CRS and 29 with ODS. Streptococcus spp prevailed in the CRS group, whereas *Staphylococcus* spp prevailed in the ODS group. Statistically significant differences between the groups were found in type of discharge, degree of edema, and presence of polyps. However, no statistical correlations were noted for presence of bacteria in the culture and endoscopic or radiological findings. **Conclusions:** ODS and CRS share some common features: ODS more often presents with purulent discharge, localized maxillary involvement, and the presence of oral pathogens, and *Staphylococcus* spp in microbial profile.

## 1. Introduction

Chronic rhinosinusitis (CRS) is a prevalent disease with quite a considerable healthcare cost burden. It is defined as the inflammation of the nose and paranasal sinuses lasting for more than 12 weeks, characterized by two or more symptoms, one of which should be either nasal blockage/congestion or nasal discharge (anterior/posterior), with or without facial pain/pressure and/or reduction in or loss of smell, and either endoscopic signs of disease or computed tomography (CT) changes [1]. Most of its pathogenic features and microbiology have been described and summarized in the European Position Paper on Rhinosinusitis and Nasal Polyps (EPOS) 2020 [1].

The usual medical treatment consists of antibiotics, intranasal steroids, and saline irrigations. In recalcitrant cases, surgical intervention, such as endoscopic sinus surgery (ESS), is often needed [1].

In contrast, odontogenic sinusitis (ODS), a subtype of sinusitis that develops from a dental origin, is a common but still frequently overlooked cause that differs in pathogenesis and management from rhinogenic sinusitis [2]. It is defined as a primarily bacterial maxillary sinusitis that can extend to other sinuses, secondary either to dental maxillary infections or following dental procedures [3,4,5]. As ODS can account for 45–75% of maxillary sinus opacifications [2,3], an odontogenic source of infection should be suspected in each case of unilateral sinusitis. Nevertheless, recently published guidelines recommend the analysis of maxillary dentition regardless of the suspected type of sinusitis [5,6].

A temporal relationship between the onset of symptoms and a dental procedure, foul smell and/or taste, congestion, rhinorrhea or retrorhinorrhea, and unilaterality of disease is suggestive of a potential ODS diagnosis, but a lack of typical dental symptoms, such as thermal pain, periapical sensitivity, or swelling, may be misleading and delay proper diagnosis and adequate treatment [6,7,8,9].

Several studies have attempted to identify the links between CRS and specific bacteria. *Staphylococcus aureus*, *Streptococcus* spp., Corynebacterium spp., and *Propionibacterium acnes* are frequently cultured, and *Haemophilus influenza*, *Escherichia coli*, Peptostreptococcus spp., Klebsiella spp., and Fusobacterium spp. are also seen, but in lower abundance [10]. It has been suggested that CRS does not result from the presence of a single species, but rather from changes in the composition or function of the microbial community [10,11].

Oral microbes live primarily as complex polymicrobial communities called biofilms or dental plaque. The microbiota of the mouth are responsible for (among others) dental caries and periodontal diseases. These oral pathogens are thought to be unable to cause disease under normal conditions, but when present in high numbers in the less well-defended sinonasal cavity they may cause distinctive infections [3,12,13].

It is established that dental pulp exhibits antimicrobial defenses against oral bacteria [14]. 

Nevertheless, caries, dental trauma, or iatrogenic procedures may lead to bacterial invasion and the colonization of the root canal space. If left untreated, it might progress to apical periodontitis with the formation of periapical lesions (PALs). Subsequent inflammatory edema in maxillary sinus (MS) and the breakdown in host defense mechanisms can progress to purulent ODS [14,15,16].

Dental conditions and procedures may encourage a large transfer of pathogens into sinonasal cavities, often modifying their physiology. Though, it seems that only select subtypes of a given species in the oral microbiota are prone to extraoral translocation with rather strain-specific adaptations, including genetic traits and host–environment interactions [10,17,18,19].

It is yet to be determined what microorganisms are more likely to cause ODS. As half of the global population has at least one tooth with PAL [15], it is still unclear what the predisposing factors are that may lead to ODS in untreated or improperly treated populations.

With more structured studies on differential diagnoses of ODS being published during the last decade, the recognition of bacteria that are more prevalent in ODS than in CRS may help clinicians to properly distinguish and treat these conditions. Drawing on the previously published scientific literature, we hypothesized that the microbiome composition in patients with CRS versus ODS would exhibit distinct differences.

## 2. Aim

The aim of this study was to compare oral and sinus microbiota in patients with ODS and CRS with clinically confirmed disease and to assess the influence of bacterial load on clinical and radiological presentations.

## 3. Materials and Methods

Patients were recruited from a tertiary referral center in 2019–2021. Adult patients scheduled for endoscopic sinus surgery due to primary diffuse chronic sinusitis in CRS cases were included in this study and prospectively followed. All patients were consulted by an otolaryngologist and dental specialist to rule out or confirm odontogenic sinusitis. Patients with previous sinus surgery, primary immunodeficiency, antibiotic intake 1 month prior to enrollment in this study, or possible coexistence of a fungal ball with chronic rhinosinusitis were excluded. 

The CT scans were evaluated and the degree of sinus opacification was scored according to the Lund–Mackay scale. The otolaryngologist assessed nasal endoscopy for mucopurulence or edema in the middle meatus or sinuses on endoscopy, whereas a dental specialist assessed pulp vitality with cold pulp testing and checked teeth with percussion, palpation, and mobility tests. 

Patients who presented with just mucosal maxillary thickening on the radiological examination ODS cases were also excluded.

The subjective symptoms were also assessed with the use of the SNOT-22 questionnaire.

Sino-Nasal Outcome Test-22 (SNOT-22) is a validated tool commonly used to assess quality of life. It includes 22 questions, and the score of each question ranges from 0 to 5, with 5 being the worst. Higher scores represent lower health-related quality of life [20].

### 3.1. Sample Collection

Patients underwent varying degrees of ESS, at least maxillary antrostomies on the affected side. All patients received a single preoperative dose of intravenous cefazolin or clindamycin in case of β-lactam hypersensitivity.

Samples were collected from all patients at the onset of surgery. At first, a standard endoscopic maxillary antrostomy (MMA) was created on the affected side. Then, the sinus was irrigated with 250 cc of normal saline (0.9% solution of NaCl) using a 30 cc syringe and a curved olive tip suction device. The aspirated rinse was collected into sterile sets at room temperature. The samples were immediately sent for culturing to the Laboratory of Microbiology, Department of Laboratory Diagnostics, Military Institute of Medicine—National Research Institute.

### 3.2. Bacterial Culture

The culture and species verification of bacteria was performed in accordance with routine diagnostic procedures at the institutional Laboratory of Microbiology, Department of Laboratory Diagnostics. Material was promptly cultured on nonselective media (Columbia agar supplemented with 5% sheep blood, McConkey agar, Chapman agar, and Sabouroud agar), as well as the selective medium for *Haemophilus* species and Schaedler agar for the cultivation of anerobic bacteria. All plates were incubated at 37 °C under a 5% CO_2_ atmosphere, except for Schaedler agar plates, which were cultured at the same temperature but under anerobic conditions. Then, mixed colonies were processed with a culture purification procedure to obtain pure isolates.

The microbes were identified by Matrix-Assisted Laser Desorption Ionization-Time Of Flight (MALDI-TOF) mass spectrometry (VITEK MS, bioMérieux, Paris, France).

Patients with prior sinus surgery, sinonasal neoplasm, autoimmune disease, and primary or acquired immunodeficiency were excluded from this study, as well as patients with a history of antimicrobial therapy one month before inclusion in the study.

### 3.3. Statistical Analysis

Statistical analysis was conducted using the Statistica 13.3 package (StatSoft Poland, Dell Statistica Partner). The quantitative data were summarized using descriptive statistics (mean and standard deviation [±SD], median and range). The distribution of each variable was tested for consistency with the normal distribution using the Kolmogorov–Smirnov test. The qualitative data are presented as percentages. To assess whether there was a statistically significant difference between groups in the number of affected sinuses, and in cases of type of discharge, degree of swelling, and presence of polyps, the Chi2 or Fisher’s exact test was performed. Moreover, to examine whether there was a statistically significant difference between the groups in the total score of the SNOT-22 questionnaire Lund–Mackay scale, the non-parametric Mann–Whitney U test was used. The significance level for the statistical tests was set at 0.05.

### 3.4. Statement of Ethics

This study was carried out in accordance with “The Code of Ethics of the World Medical Association (Declaration of Helsinki)” for experiments involving humans.

It was approved by the Ethics Committee of the Military Institute of Medicine on 16 October 2019 (protocol No 43/WIM/2019) and written informed consent was obtained from each participant.

## 4. Results

The study group consisted of 84 patients, of whom 50 (59.5%) were men and 34 (40.5%) women. The age range was from 23 to 77 years, the mean age was 47.7 ± 12.8 years, and the median age 45 years. The BMI range was from 17.2 to 40.5 [kg/m^2^], mean BMI 27.5 ± 5.1 [kg/m^2^], and median 27.4 [kg/m^2^].

A total of 55 patients were diagnosed with non-odontogenic lesions (110 sinuses were assessed) while 29 were diagnosed with odontogenic lesions (32 sinuses were assessed). The characteristics of the group of patients with non-odontogenic lesions were 32 men (58.2% of the group) and 23 women (41.8% of the group), age range from 25 to 70 years, mean age 47.8 ±12.3 years, median age 45 years, BMI range from 17.4 to 39.2 [kg/m^2^], mean BMI 27.6 ± 4.6 [kg/m^2^], and median BMI 27.4 [kg/m^2^]. The characteristics of the group of patients with odontogenic lesions were 18 men (62.1% of the group) and 11 women (37.9% of the group), age range from 29 to 77 years, mean age 47.3 ±13.8 years, median age 44 years, BMI range from 17.2 to 40.5 [kg/m^2^], mean BMI 27.1 ± 6.1 [kg/m^2^], and median BMI 26.1 [kg/m^2^]. There were no statistically significant differences due to the age, sex, and BMI between the subgroups, *p* = 0.751, *p* = 0.780, and *p* = 0.432, respectively. The data are compared in Table 1.

**Figure 1:**
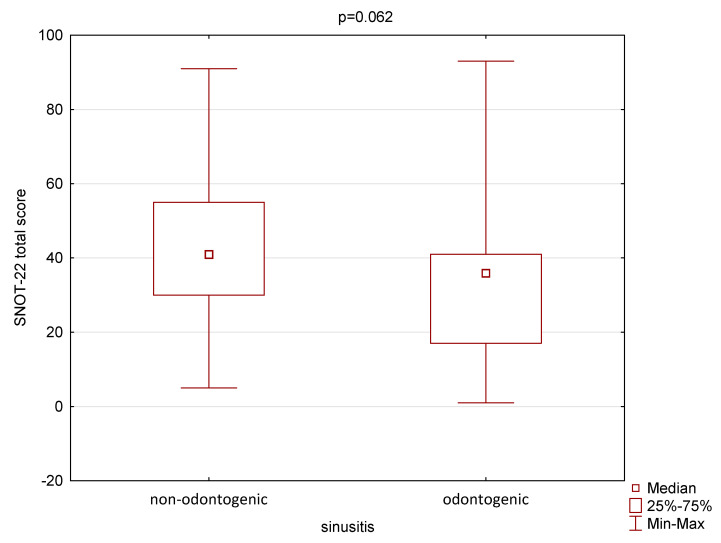
The mean total score of the Sino-Nasal Outcome Test-22 (SNOT-22) questionnaire in the group of CRS and ODS patients. CRS—chronic sinusitis; ODS—odontogenic sinusitis.

The mean total score of the SNOT-22 questionnaire in the group of CRS patients was 43.2 ± 21.5, median 41, and ranged from 5 to 93. In the group of subjects with ODS, the mean total score was 33.9 ± 23.6, median 36, and range from 51 to 93. The non-parametric Mann–Whitney U test did not show statistically significant differences between the groups (*p* = 0.062). The results are presented in Figure 1.

**Figure 2:**
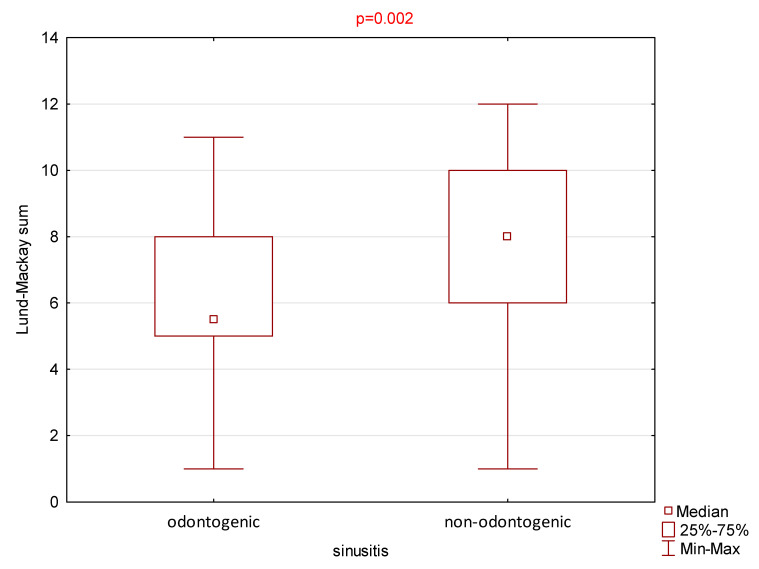
The mean total score of the Lund–Mackay scale in the group of subjects with odontogenic sinusitis (ODS) and non-odontogenic sinusitis (CRS).

The mean total score of the Lund–Mackay scale in the group of subjects with CRS was 7.9 ± 2.7, median 8, and score range from 1 to 12. In the group of subjects with ODS, the mean total score was 6.3 ± 2.4, median 5.5, and range from 1 to 11. Statistically significant differences were found between the two patient groups (*p* = 0.002 Mann–Whitney U test). The results are presented in Figure 2.

In each subgroup of patients, material was collected from the sinuses to be assessed for microbiological purposes. Initially, only the presence of a bacteria culture was checked. In the subgroup of patients with non-odontogenic lesions, the presence of bacteria was confirmed in a higher number of sinuses than in the subgroup with odontogenic lesions (73 [66.4%] vs. 19 [59.4%]), but the result is not statistically significant (*p* = 0.466)

Afterwards, the type of bacteria found in the culture was considered. In the CRS group, the most frequent bacterial species was *Streptococcus*, which was found in a quarter of the cases (8 out of 32 cases). On the other hand, in the ODS subgroup, the most common bacteria were *Staphylococcus* sp. found in 28 (25.5%) sinuses and *Staphylococcus aureus* in 20 (18.2%) sinuses. In the non-odontogenic lesion subgroup, the following bacteria were found once in different sinuses (3.1%): *Prevotella nigrescens*, *Cutibacterium avidum*, *Hafnia alvei*, *Prevotella buccae*, *Fusobacterium nucleatum*, *Streptococcus anginosus*, *Raoultella ornithinolytica*, *Staphylococcus epidermidis*, *Staphylococcus aureus*, *Streptococcus constellatus* together with *Streptococcus viridans*, and *Staphylococcus aureus* together with *Streptococcus pyogenes*. Meanwhile, in the odontogenic lesion group, the following bacteria had an incidence of two sinuses (1.82%): *Klebsiella pneumoniae*, *Escherichia coli*, *Streptococcus pneumoniae*, *Proteus mirabilis*, *Enterobacter aerogenes* with *Proteus mirabilis*, and *Citrobacter koseri* with *Staphylococcus* spp. The following bacteria had an incidence of one sinus: *Pseudomonas aeruginosa*, *Citrobacter koseri*, *Klebsiella oxytoca*, *Kocuria*, *Enterococcus faecalis* with *Staphylococcus* spp., *Citrobacter koseri* with *Klebsiella pneumoniae and Staphylococcus aureus*, *Pseudomonas aeruginosa* with *Escherichia coli*, *Staphylococcus aureus* with *Klebsiella pneumoniae*, *Staphylococcus aureus* with *Escherichia coli*, and *Klebsiella pneumoniae* with *Escherichia coli*. Statistically significant differences were found between the two groups.

Statistically significant differences were noted between the two patient groups in case of type of discharge, degree of edema, and presence of polyps. Purulent discharge occurred more often in the group of patients with odontogenic lesions (50.0% vs. 18.2%), as well as grade 2 swelling (64.6% vs. 84.4%), while polyps more occurred often in the non-odontogenic sinusitis group (40.0% vs. 4.1%, Table 2). However, no statistically significant correlations were found between the presence of a bacteria culture and type of discharge and swelling in any of the groups (Table 3).

**Figure 3:**
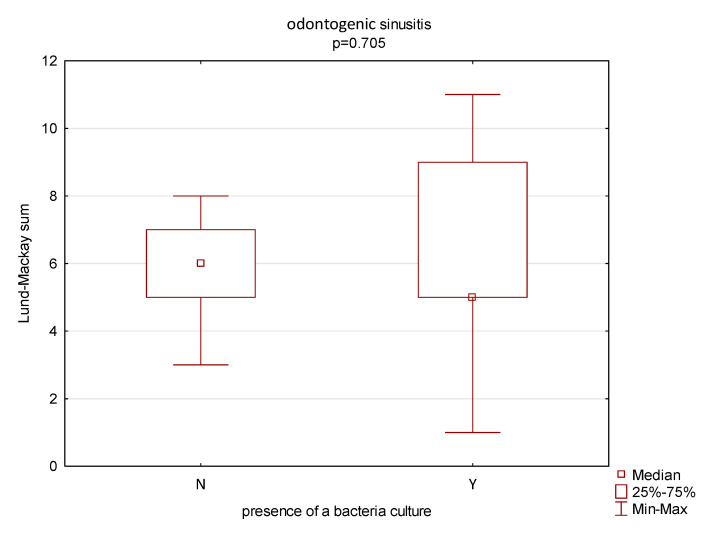
Correlation between the extension of radiological lesions (Lund–Mackay scale) and the presence of a bacteria culture in the group of patients with odontogenic sinusitis. Y—bacteria present; N—bacteria not detected.

**Figure 4:**
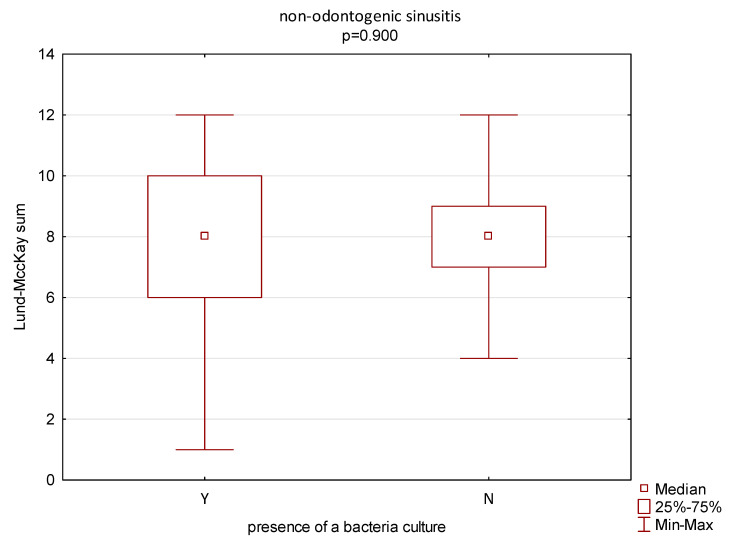
Correlation between the extension of radiological lesions (Lund–Mackay scale) and the presence of a bacteria culture in the group of patients with non-odontogenic sinusitis. Y—bacteria present; N—bacteria not detected.

There were also no statistically significant differences in the extension of radiological lesions on the Lund–Mackay scale or the presence of a bacteria culture in any of the groups (*p* = 0.705 and *p* = 0.900, respectively) in the CRS and ODS groups (Figure 3 and Figure 4).

## 5. Discussion

This study compared the microbiological profiles of ODS and CRS to enhance our understanding of bacterial composition in these two distinct conditions. While both types of sinusitis exhibit overlapping symptoms, the results of the study underscore important differences in the bacterial composition, radiographic findings, and clinical presentation, which might have implications for both diagnosis and treatment strategies [21].

The microbiological analysis revealed that while both ODS and CRS involve diverse bacterial communities, significant differences exist in the types and distribution of bacterial species between these two groups. In the CRS group, *Streptococcus* species were the most prevalent, aligning with the previous literature that identifies *Streptococcus pneumoniae* and *Streptococcus viridans* as common pathogens in sinusitis originating from the respiratory tract [1,8]. This predominance of *Streptococcus* in non-odontogenic sinusitis is consistent with the hypothesis that CRS often results from respiratory pathogens that colonize the sinonasal cavity and subsequently trigger chronic inflammation.

In contrast, the ODS group exhibited a higher prevalence of *Staphylococcus* species, including *Staphylococcus aureus*, which aligns with the findings from other studies that have reported similar bacteria in odontogenic infections [5,10]. The presence of *S. aureus* and other oral-associated pathogens in ODS suggests that these bacteria may enter the sinus cavity following dental infections or procedures, potentially facilitated by anatomical pathways [2,13].

Several bacterial species typically found in the oral microbiome, such as *Prevotella* spp., *Fusobacterium* spp., and *Peptostreptococcus* spp., were identified in the ODS group. These anaerobes are known for their role in dental and periodontal diseases and are rarely implicated in non-odontogenic sinusitis. The similarity between the germs in the oral cavity and those in ODS is observed in many studies, because of the high variation in the flora of periapical infections involved in the etiology of these infections. Intraradicular bacteria that may cause secondary periapical lesions are among *Streptococcus*, *Propionibacterium*. But causative microorganisms of periradicular pathology might be difficult to identify, as the bacteria can be extraradicular and diffuse to the periapical area [3,7,12,22,23].

The presented study’s results also highlight significant differences in the clinical presentation and radiographic findings between ODS and CRS. While the SNOT-22 scores did not significantly differ between groups, patients with non-odontogenic lesions had higher Lund–Mackay scores, suggesting that CRS involves more extensive radiological abnormalities (mean score: 7.9 vs. 6.3). This is consistent with the previous research indicating that CRS is often associated with widespread sinonasal inflammation and more likely present bilaterally, while ODS tends to be more localized, frequently affecting only the maxillary sinus on the affected side [3,4].

Cone beam computed tomography was proved to better correlate with the periapical lesion’s status and affected maxillary sinus than panoramic radiography, which makes it the radiological examination of choice, whenever accessible [24,25,26,27].

Additionally, patients with ODS were more likely to exhibit purulent nasal discharge and grade 2 mucosal swelling, whereas polyps were more commonly observed in the CRS group [7].

Misdiagnosis of ODS as CRS can lead to suboptimal treatment, as traditional therapies for CRS, such as nasal corticosteroids and saline irrigations, may not effectively target the bacteria typically involved in odontogenic infections [28,29]. Given the prevalence of bacteria such as *Staphylococcus aureus* and anaerobes in ODS, antibiotic regimens tailored to cover these pathogens may be more appropriate for managing odontogenic infections. Furthermore, addressing the underlying dental source of infection is crucial to achieving successful outcomes in ODS, as untreated dental infections can perpetuate sinus involvement [14,20,21].

One of the more significant findings in our study is the quantification of CRS and ODS differences through a direct, statistically significant comparison of the Lund–Mackay scale scores (*p* = 0.002), which has not always been a primary focus in previous studies. This could add value to refining diagnostic criteria for ODS and recognizing its clinical features [2,30,31].

The use of the Lund–Mackay scale and a careful evaluation of bacterial cultures may assist clinicians in distinguishing between ODS and CRS. For example, the presence of oral-associated anaerobes in sinus cultures, along with unilateral maxillary sinus opacification on imaging, should prompt the consideration of an odontogenic origin, even in the absence of overt dental symptoms.

The presented research also emphasizes the relationship between symptom severity and bacterial culture, which directly ties symptomatology with the microbial findings. This may have important implications for clinical management, as raising the suspicion of ODS based on clinical presentation and bacterial profiles can help guide more effective treatments.

From a clinical perspective, methods of sample collection, patient demographics, or microbiological techniques could influence all the outcomes published so far.

A study by Saibene et al. explored microbiological differences between ODS and CRS, focusing on the types of bacteria isolated from patients with these conditions. The most common pathogens in ODS were staphylococci, particularly *S. aureus* (though prevalent also in CRS), along with anaerobes, such as *Peptostreptococcus spp.*, *Fusobacterium nucleatum*, and *Dialister pneumosintes* [4]. Polymicrobial growth was also stated by Yassin Kassab et al. In their study, the following bacteria were significantly more likely in ODS compared to CRS: mixed anaerobes, *Fusobacterium spp.*, *Eienella corrodens*, *Streptococcus intermedius*, *Streptococcus anginosus*, and *Streptococcus constellatus*, while *Pseudomonas aeruginosa* and *Staphylococcus aureus* were inversely correlated with ODS [3].

A study by Puglisi et al. found that *S. aureus* was a major pathogen in both ODS and CRS, but they also noted that ODS had higher rates of *Propionibacterium acnes* and *Prevotella* spp. [22]. This is in accordance with our results. The presence of additional species, such as *Klebsiella pneumoniae*, *Pseudomonas aeruginosa*, and *Enterococcus faecalis* in the ODS group, albeit in lower prevalence, further supports the notion that ODS has more diverse microbiological diversity compared to CRS [8,32].

The recent study by Wu et al. suggested that dominant bacteria from the nasal and oral cavities (such as *Fusobacterium* spp. or *Propionibacterium acnes*) closely compete and might act as a deterrent to pathogenic bacteria in CRS populations, whereas dominant pathogens in the nasal and oral cavities of ODS patients interact with each other, which might promote the development of disease [33]. The presence of anerobic bacteria in the nasal microbiome of ODS patients may indicate potential tissue hypoxia or oxygen limitation within the mucus or bacterial biofilm in ODS patients, allowing anerobic bacteria to survive [33].

The identification of *S. aureus* as a predominant pathogen in ODS underscores the importance of considering a dental origin in cases of chronic unilateral sinusitis. While many cases of CRS are also polymicrobial, dominated by commensal bacteria, ODS is more likely to be dominated by specific pathogens, including *S. aureus*, and occasionally other oral-origin species, like *Klebsiella pneumoniae* and *Pseudomonas aeruginosa*.

## 6. Limitations

Several limitations should be noted. First, this study’s sample size, though sufficient to detect significant differences in some parameters, may limit the generalizability of these findings. Additionally, this study relied on culture-based methods for bacterial identification, which may underestimate the diversity of bacteria present, particularly anaerobes that are difficult to culture. Advanced molecular techniques, such as 16S rRNA sequencing, could provide a more comprehensive picture of the microbial communities in both ODS and CRS, potentially revealing additional species and clarifying the microbial diversity of each condition. Nevertheless, 16S rRNA sequencing can show dead species that are no longer present as a life part of sinus microbiome, making that method imperfect as well.

Future research should also explore the role of biofilm formation in ODS and CRS, as biofilms may contribute to the persistence of infection and resistance to treatment. Further studies are needed to identify the risk factors that predispose individuals with dental infections to develop ODS, as well as to investigate the efficacy of targeted antibiotic therapies in eradicating the specific pathogens involved in ODS.

## 7. Conclusions

In summary, while there is some overlap in the microbiological findings between ODS and CRS, these conditions exhibit distinct microbiological and clinical characteristics. ODS is more likely to involve bacteria associated with the oral cavity, such as *Staphylococcus aureus*, and anerobic pathogens with a broader range of species. While both conditions share some common pathogens, ODS additionally presents with features such as purulent discharge and localized maxillary involvement and a distinct microbial profile. The detailed microbiological comparison, the inclusion of both clinical and radiological factors, and the rigorous sampling methods strengthen this research in this field. Understanding the differences in ODS and CRS is critical for ensuring optimal treatment and avoiding unnecessary broad-spectrum antibiotics, particularly in a clinical environment that requires precision due to growing antibiotic resistance.

## Figures and Tables

**Table 1:** Descriptive statistics of demographic features between patients with odontogenic and non-odontogenic sinusitis. BMI—body mass index; SNOT-22—Sino-Nasal Outcome Test 22; SD—standard deviation.

	Non-Odontogenic		
	age	BMI	SNOT-22
**Mean ± SD**	47.8 ± 12.3	27.6 ± 4.6	43.2 ± 21.5
**Median** **(Min–Max)**	45.0 (25.0–70.0)	27.4 (17.4–39.2)	41.0 (5.0–91.0)
	**Odontogenic**		
**Mean ± SD**	47.3 ± 13.8	27.1 ± 6.1	33.9 ± 23.6
**Median** **(Min–Max)**	44.0 (23.0–77.0)	26.1 (17.2–40.5)	36 (1–93)

**Table 2:** Modified Lund–Kennedy endoscopic score and the level of statistical significance (*p*-value) between odontogenic sinusitis (ODS) and non-odontogenic sinusitis (CRS) patients.

	Discharge n (%)			*p*-Value
	None	Clear, thin	Purulent	0.001
ODS	2 (6.3%)	14 (43.7%)	16 (50.0%)	
CRS	3 (2.7%)	87 (79.1%)	20 (18.2%)	
	**Edema n (%)**			0.007
	Grade 0	Grade 1	Grade 2	
ODS	2 (6.3%)	3 (9.3%)	27 (84.4%)	
CRS	0 (0.0%)	39 (35.5%)	71 (64.6%)	
	**Polyps n (%)**			*p* < 0.001
	None	Restricted to the middle meatus	Beyond the middle meatus	
ODS	26 (81.3%)	5 (15.6%)	1 (3.1%)	
CRS	54 (49.1%)	12 (10.9%)	44 (40.0%)	

**Table 3:** The correlation of edema and discharge and the presence of bacteria in culture from odontogenic and non-odontogenic sinusitis patients. *p*-value—the level of statistical significance.

Presence of Bacteria in Culture (Y—Yes/N—No)	Edema			*p*-Value	Discharge			*p*-Value
**odontogenic**								
	0	1	2		0	1	2	
Y	2 (10.5%)	17 (89.5%)	0 (0.0%)	0.227	1 (5.3%)	8 (42.1%)	10 52.6%	0.920
N	0 (0.0%)	13 (100.0%)	0 (0.0%)		1 (7.7%)	6 (46.2%)	6 46.2%	
**non-odontogenic**								
Y	0 (0.0%)	22 (30.1%)	51 (69.9%)	0.152	2 (2.7%)	56 (76.7%)	15 (20.6%)	0.662
N	0 (0.0%)	17 (46.0%)	20 (54.0%)		1 (2.7%)	31 (83.8%)	5 (13.5%)	

## Data Availability

The raw data supporting the conclusions of this article will be made available by the corresponding author on request.

## Abbreviations

ODS	Odontogenic sinusitis
CT	Computed tomography
CBCT	Cone beam computed tomography
PAL	Periapical lesion
RCT	Root-canal treatment
ESS	Endoscopic sinus surgery
MS	Maxillary sinus
MMA	Middle meatal antrostomy
CRS	Chronic rhinosinusitis
